# Acute hemorrhage with huge extraperitoneal hematoma following transrectal prostate biopsy: indication for emergency prostatectomy – a case report

**DOI:** 10.1093/jscr/rjag194

**Published:** 2026-03-26

**Authors:** Toni Franz, Ulrich Stallkamp, Thomas Lingscheidt, Lars-Christian Horn, Jens-Uwe Stolzenburg

**Affiliations:** Department of Urology, University of Leipzig, Liebigstraße 20a, 04103, Leipzig, Saxony, Germany; Carl-von-Basedow-Klinikum Saalekreis gGmbH, Weiße Mauer 52, 06217, Merseburg, Saxony-Anhalt, Germany; University of Leipzig, Institute of Pathology, Liebigstraße 26, 04103, Leipzig, Saxony, Germany; University of Leipzig, Institute of Pathology, Liebigstraße 26, 04103, Leipzig, Saxony, Germany; Department of Urology, University of Leipzig, Liebigstraße 20a, 04103, Leipzig, Saxony, Germany

**Keywords:** emergency robotic surgery, extraperitoneal hematoma, hemorrhagic complication, prostate biopsy, radical prostatectomy

## Abstract

Prostate biopsy is generally safe, and extraperitoneal hematomas are extremely rare. We report the first known case of ongoing post-biopsy bleeding managed with emergency robot-assisted radical prostatectomy. A 77-year-old man developed acute hemodynamic instability several hours after a transrectal MRI/US-fusion biopsy. Computed tomography revealed a large left-sided extraperitoneal hematoma displacing the bladder without arterial extravasation. Despite resuscitation, hemoglobin continued to decrease, suggesting persistent venous bleeding. Preliminary histology confirmed well-differentiated acinar adenocarcinoma. Given hematoma size, instability, and confirmed malignancy, interdisciplinary consensus favored surgical management. Within 24 h, the patient underwent extraperitoneal bilateral nerve-sparing radical prostatectomy with complete hematoma evacuation. The procedure was uncomplicated, recovery stable, continence satisfactory, and final pathology showed pT2c Gleason 3 + 3 = 6 cancer with negative margins. This case underscores the need for vigilance regarding atypical post-biopsy symptoms and shows that emergency prostatectomy may offer both hemostasis and definitive oncologic treatment.

## Introduction

Prostate cancer remains one of the most frequently diagnosed malignancies in men, and tissue diagnosis through prostate biopsy is essential for risk stratification and treatment planning [1]. Guideline-based indications include persistently elevated prostate-specific antigen (PSA) levels, suspicious PSA kinetics, or abnormalities on digital rectal examination [2, 3]. Although generally safe, prostate biopsy carries risks. While minor complications such as self-limiting hematuria, hematospermia, and rectal bleeding are relatively common, severe complications, including large retroperitoneal or extraperitoneal hematomas, are exceedingly rare but potentially life-threatening [4–14]. The management is typically conservative, although interventional radiology or surgery may be required. To the best of our knowledge, immediate hematoma evacuation combined with simultaneous radical prostatectomy has not been previously described. We report the first case of robot-assisted endoscopic extraperitoneal bilateral nerve-sparing radical prostatectomy with hematoma evacuation performed as an emergency procedure within 24 h of biopsy.

## Case report

**Figure 1:**
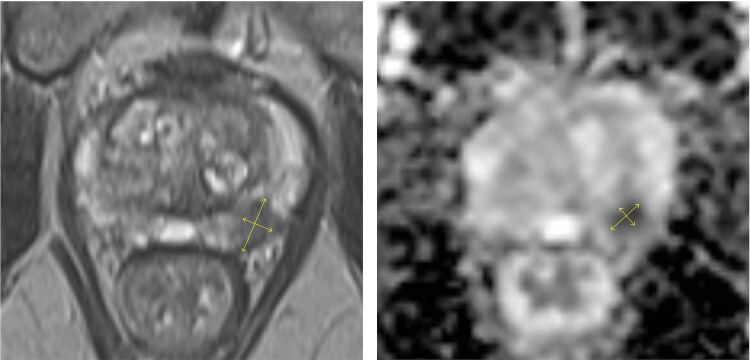
Multiparametric MRI of the prostate showing an 11 × 6 mm PI-RADS 4 lesion in the mid-third of the left dorsolateral peripheral zone with marked hypointensity on T2-weighted and diffusion-weighted images.

A 77-year-old male without relevant comorbidities and not on anticoagulant therapy was referred for prostate biopsy after PSA increased from 4.5 to 8.1 ng/ml over 6 months. Multiparametric magnetic resonance imaging (MRI) demonstrated moderate benign prostatic hyperplasia (5.0 × 3.7 × 4.1 cm; 38 ml) and a PI-RADS 4 lesion (11 × 6 mm) in the mid-third of the left peripheral zone, dorsolaterally. The lesion showed hyperintensity on diffusion-weighted imaging (DWI) and hypointensity on T2-weighted sequences, consistent with restricted diffusion (Fig. 1). A 15-core systematic transrectal MRI/ultrasound fusion biopsy was performed after intravenous administration of ertapenem (1 g). The patient experienced unusually intense pain during the procedure that regressed spontaneously. A post-biopsy rectal examination revealed no evidence of significant bleeding.

**Figure 2:**
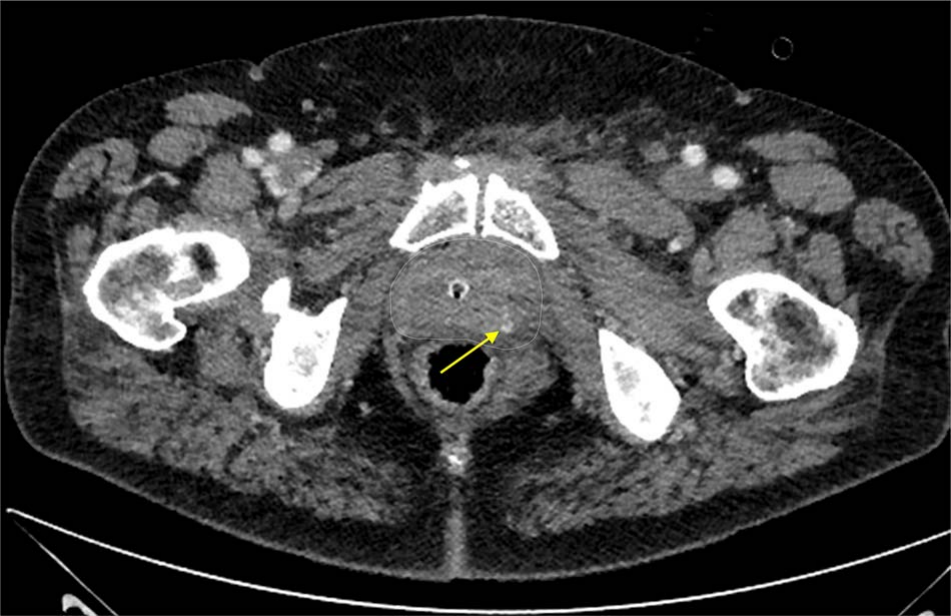
Contrast-enhanced CT scan of the prostate showing focal extravasation in the left dorsolateral midgland, suggesting slow venous oozing. The prostate contour is marked by a dashed line.

**Figure 3:**
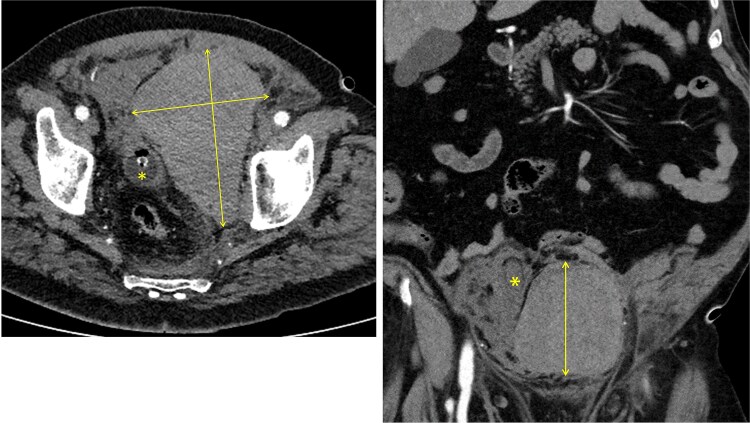
Left: axial CT showing a 14 × 9.5 cm extraperitoneal hematoma with urinary catheter balloon marked by an asterisk. Right: coronal CT showing an 11 × 9.5 cm extraperitoneal hematoma with the urinary bladder displaced to the right, indicated by an asterisk.

Approximately 6 h after biopsy, the patient was admitted to the emergency department with deterioration, abdominal pain, hypotension, and tachycardia. On arrival, blood pressure was 80/40 mmHg and tachycardia (124 bpm). Laboratory tests revealed leukocytosis (21.9 × 10^9^/l), elevated creatinine (132 μmol/l, eGFR 44 ml/min/1.73 m^2^), and hemoglobin 6.9 mmol/l. Emergency computed tomography (CT) revealed a large left-sided extraperitoneal hematoma (14 × 11 × 9.5 cm) that displaced the bladder, with capsular venous bleeding but no major arterial source (Figs 2 and 3). Hemodynamic stabilization was achieved with fluid resuscitation and norepinephrine at 0.1 μg/kg/min. Cefotaxime was initiated empirically.

The following day, the hemoglobin level dropped further to 5.3 mmol/l, indicating ongoing bleeding. Expedited pathology revealed bilateral well-differentiated acinar adenocarcinoma in 4 of the 15 cores, with up to 80% tumor involvement. Although conservative management is typically considered the first-line strategy for post-biopsy hematoma, this approach is deemed insufficient. Given the persistent bleeding, large hematoma size, organ displacement, and histological confirmation of cancer, surgical revision was favored. Delayed intervention increases the risk of infection and technical difficulty.

**Figure 4:**
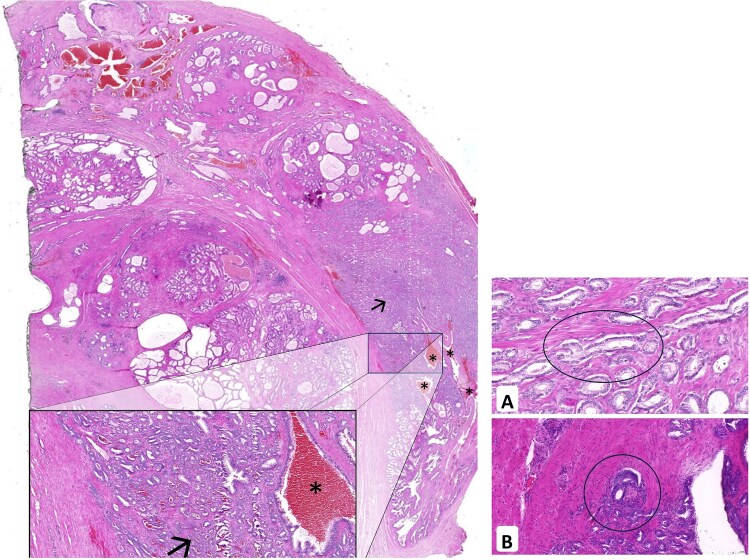
Left: histopathology of the prostatectomy specimen showing well-differentiated acinar adenocarcinoma (Gleason 3 + 3 = 6, pT2c) with L1 and Pn1; surgical margins are tumor-free (R0). Fresh hemorrhagic changes are noted dorsolaterally. Right: L1 (A) and Pn1 (B) highlighted.

Within 24 h of biopsy, the patient underwent robot-assisted endoscopic extraperitoneal bilateral nerve-sparing radical prostatectomy with hematoma evacuation. Intraoperatively, a large fresh hematoma was identified in the left extraperitoneal space and was removed completely via suction. No definitive source of extraprostatic bleeding was identified. An uneventful radical prostatectomy was performed. Recovery was smooth: the ultrasound was normal, the drain was removed on day 2, and the catheter was removed on day 6 after cystographic confirmation of an intact anastomosis. Histopathology revealed well-differentiated acinar adenocarcinoma (Gleason 3 + 3 = 6), pathological stage pT2c, with lymphatic (L1) and perineural invasion (Pn1). All the margins were negative (R0). Hemorrhagic changes were noted near the bladder neck and dorsolateral region (Fig. 4). At one month, the patient fully recovered and reported continence with only one safety pad at night.

## Discussion

Transrectal prostate biopsy is widely used and generally safe. Most complications are minor and self-limiting, and include hematuria, hematospermia, rectal bleeding, and lower urinary tract symptoms (LUTS) [4]. Hematomas following pelvic trauma or intervention typically accumulate in the prevesical (Retzius) space, a compartment between the bladder and anterior abdominal wall. This region is connected to the periprostatic venous plexus and inferior vesical artery, allowing fluid extension, whereas Denonvilliers’ fascia limits spread posteriorly. These hematomas may compress adjacent structures such as the bladder or sigmoid colon, causing pelvic pain or urinary urgency. Small extraperitoneal hematomas are usually managed conservatively without surgical exploration [5–13]. Severe hemorrhagic events are rare but potentially life-threatening.

Our case illustrates a rare and dramatic complication: ongoing bleeding with a large extraperitoneal hematoma initially associated with hemodynamic instability and hemoglobin decline, ultimately necessitating emergency surgical management or evaluation with interventional radiology [6, 14].

Only a few similar cases have been reported to date. Choyke *et al*. first described a prevesical hematoma after transrectal biopsy in 1986 [7] that was managed conservatively [5, 7–13]. Kaneko *et al.* reported a 62-year-old patient with massive retroperitoneal hemorrhage from the prostatic artery who was treated successfully with transarterial embolization (TAE) [6]. Malik *et al*. described severe rectal bleeding controlled by endoscopic clipping, demonstrating a minimally invasive therapeutic option [14]. However, none of these reports involved simultaneous hematoma evacuation and radical prostatectomy in an acute setting.

In the present case, the therapeutic decision was based on an intensive interdisciplinary evaluation, including conservative management, less invasive interventional radiological approaches such as transcatheter arterial embolization (TAE), and surgical intervention.

Furthermore, the hematoma’s substantial size with organ displacement, ongoing hemoglobin decline, and risk of secondary infection or fibrotic organization argued against continued conservative management. The patient’s physiological status and confirmed diagnosis of acinar adenocarcinoma of the prostate provided the opportunity to perform a procedure that was both hemostatic and oncologically definitive. After thorough evaluation of the clinical and imaging findings and in close agreement with the patient, an emergency robot-assisted extraperitoneal nerve-sparing radical prostatectomy with hematoma evacuation was undertaken as the most appropriate and timely intervention.

Generally, even in hemodynamically unstable patients, TAE can be regarded as the first-line modality for hemorrhage control.

To the best of our knowledge, this is the first reported case of robot-assisted endoscopic hematoma evacuation combined with bilateral nerve-sparing extraperitoneal radical prostatectomy performed as an emergency procedure within 24 h of biopsy. This case highlights the necessity of clinical vigilance following prostate biopsy, especially in elderly patients or those with atypical symptoms such as persistent pain or signs of hemodynamic instability. Rapid imaging, interdisciplinary decision making, and readiness for surgical management are crucial in high-risk scenarios. In unstable patients with large hematomas, conservative management may not be sufficient. Surgical intervention not only stabilized our patient but also provided curative cancer treatment.

### Limitations

This case report is inherently limited by its single-patient nature, which restricts its generalizability. The decision to perform emergency radical prostatectomy reflects a unique clinical scenario and may not be applicable to all patients with postbiopsy hemorrhage. The exact source of bleeding was localized only by CT imaging and could not be definitively identified without angiography. Additionally, long-term functional and oncological outcomes beyond the early postoperative period are not available.

## Conclusion

In conclusion, this case demonstrates that severe extraperitoneal hemorrhage following transrectal prostate biopsy, although exceptionally rare, can progress rapidly and requires immediate intervention. Despite stabilization efforts, the combination of hemodynamic instability, large hematoma size with organ displacement, and ongoing bleeding rendered conservative management insufficient. This case underscores the importance of promptly recognizing atypical post-biopsy deterioration, rapid imaging, and interdisciplinary evaluation. In selected patients, particularly when malignancy is already confirmed, and hematoma threatens clinical stability, early surgical intervention may represent a safe and effective alternative to conservative or radiological management strategies.
